# Genotoxic Effects of Aluminum Chloride and Their Relationship with N-Nitroso-N-Methylurea (NMU)-Induced Breast Cancer in Sprague Dawley Rats

**DOI:** 10.3390/toxics8020031

**Published:** 2020-04-20

**Authors:** Alejandro Monserrat García-Alegría, Agustín Gómez-Álvarez, Iván Anduro-Corona, Armando Burgos-Hernández, Eduardo Ruíz-Bustos, Rafael Canett-Romero, Humberto González-Ríos, José Guillermo López-Cervantes, Karen Lillian Rodríguez-Martínez, Humberto Astiazaran-Garcia

**Affiliations:** 1 Programa de Doctorado en Ciencias Químico Biológicas y de la Salud, Universidad de Sonora, 83000 Hermosillo, Sonora, Mexico; eduardo.ruiz@unison.mx; 2 Departamento de Ciencias Químico Biológicas, Universidad de Sonora, 83000 Hermosillo, Sonora, Mexico; 3 Departamento de Ingeniería Química y Metalurgia, Universidad de Sonora, 83000 Hermosillo, Sonora, Mexico; agustin.gomez@unison.mx; 4 Centro de Investigación en Alimentación y Desarrollo, AC, 83304 Hermosillo, Sonora, Mexico; ivan.anduro@ciad.mx (I.A.-C.); hugory@ciad.mx (H.G.-R.); 5 Departamento de Investigación y Posgrado en Alimentos, Universidad de Sonora, 83000 Hermosillo, Sonora, Mexico; armando.burgos@unison.mx (A.B.-H.); rafacanett@gmail.com (R.C.-R.); 6 Departamento de Medicina y Ciencias de la Salud, Universidad de Sonora, 83000 Hermosillo, Sonora, Mexico; guillermo.lopez@unison.mx; 7 Licenciatura en Nutrición Humana, Universidad Estatal de Sonora, Unidad Académica Hermosillo, 83100 Hermosillo, Sonora, Mexico; karenroma.ues@gmail.com

**Keywords:** genotoxicity, aluminum chloride, rats

## Abstract

Recently, soluble forms of aluminum for human use or consumption have been determined to be potentially toxic due to their association with hepatic, neurological, hematological, neoplastic, and bone conditions. This study aims to assess the genotoxic effect of aluminum chloride on genomic instability associated with the onset of N-nitroso-N-methylurea (NMU)-induced breast cancer in Sprague Dawley rats. The dietary behavior of the rats was assessed, and the concentration of aluminum in the mammary glands was determined using atomic absorption spectroscopy. Genomic instability was determined in the histological sections of mammary glands stained with hematoxylin and eosin. Moreover, micronucleus in peripheral blood and comet assays were performed. The results of dietary behavior evaluation indicated no significant differences between the experimental treatments. However, aluminum concentration in breast tissues was high in the +2000Al/−NMU treatment. This experimental treatment caused moderate intraductal cell proliferation, lymph node hyperplasia, and serous gland adenoma. Furthermore, micronucleus and comet test results revealed that +2000Al/−NMU led to a genotoxic effect after a 10-day exposure and the damage was more evident after a 15-day exposure. Therefore, in conclusion, genomic instability is present and the experimental conditions assessed are not associated with breast cancer.

## 1. Introduction

Aside from the multiple industrial applications of aluminum, it has applications in different fields including a few uses related to human consumption such as a flocculant in drinking water purification processes, as an adjuvant in vaccine preparation, and as an additive in beverages, foods (e.g., sweets and cheeses), antiperspirants and deodorants, among others [1,2,3,4,5,6,7]. However, the soluble forms of aluminum are considered potentially toxic because of their high water solubility and genotoxic capacity and because of our exposure to these soluble forms at subacute and chronic levels on a daily basis [6,8,9]. Recent estimates from the United States denote that middle-class young adults consume an average of 105–150 mg of aluminum per day in food and drinks, indicating that individuals are constantly exposed to the frequent use or consumption of different forms of aluminum [4,5,10]. Genomic instability is an essential prerequisite for the generation of multiple carcinogenesis-related alterations and mutations [11,12]. Therefore, trace (essential) elements, metals, and heavy metals are associated with breast cancer [13,14,15,16] either because cancer decreases the levels of some trace metals or because the metals cause or are associated with the genesis of cancer [2,17]. One of these trace metals is arsenic, which is associated with genomic instability, lung cancer [18], and deficient manganese metabolism, causing mitotic deregulation associated with genomic instability in humans [19]. Iron, another trace metal, is also associated with the development of genomic instability and liver, lung, and intestinal cancers in experimental rats [20]. Moreover, copper has reportedly been linked to genomic instability, which is related to breast cancer in rats [21], whereas cadmium is related to breast cancer in women worldwide [22]. Furthermore, aluminum chloride is being investigated for its potential relationship with neurological, hepatic, bone, and hematological conditions as well as with breast cancer [23,24,25,26,27]. Therefore, we assessed the genotoxic effects of aluminum concentration in Sprague Dawley rats and determined whether a link could be established with N-nitroso-N-methylurea (NMU)-induced breast cancer. This will aid in the development of new strategies for addressing exposure due to the use and/or consumption of soluble forms of aluminum as well as in the elucidation of their genotoxic hazards and relationship with the genesis of breast cancer in humans.

## 2. Materials and Methods

### 2.1. Chemicals

NMU N1517-1G (lot no. SLBF6813V) reagent was purchased from Sigma Chemical Co. (St. Louis, MO, USA), TRIzol^TM^ reagent was acquired from Life Technologies (Thermo Fisher Scientific, cat. no. 1596026, (Waltham, MA, USA), and the QuantiTect Reverse Transcription Kit manufactured by Qiagen (cat. no. 205311), (Germantown, MD, USA) was obtained. In addition, the TaqMan gene expression assays were purchased from Applied Biosystems (cat. nos. 4331182 and 4308313), and aluminum chloride (AlCl_3_) was procured from Sigma Chemical Co. (St. Louis, MO, USA). All other chemical products were of analytical grade.

### 2.2. Experimental Animals

We selected 32 female Sprague Dawley rats (weight between 180 and 220 g) from the biotery at the Food Sciences Graduate Studies and Research Department at the University of Sonora.

### 2.3. Experimental Design (Treatments)

Groups of eight rats were randomly assigned to one of the following experimental treatments: treatment A rats not fed with aluminum and with no breast cancer induction (negative control) (−Al/−NMU); treatment B rats fed with 2000 mg/L aluminum (AlCl_3_) and breast cancer induced by NMU (+2000Al/+NMU); treatment C rats fed with 2000 mg/L aluminum (AlCl_3_) with no breast cancer induction (+2000Al/−NMU); and treatment D rats not fed with aluminum and breast cancer induced by NMU (positive control) (−Al/+NMU). Rats not fed with aluminum (AlCl_3_) and with no breast cancer induction (NMU) were treated with 0.98% physiological saline to match the experimental conditions. Rats were intragastrically (gavage) fed 1 mL AlCl_3_ 5 days/week for 90 days, whereas NMU was intraperitoneally administered at 50 and 70 days of age [28,29]. A volume of 1 mL of AlCl_3_ solution with a concentration of 2000 mg/L is equivalent to administering 2 mg of aluminum to rats, considering that the mean weight of rats is 0.2 kg. This is equivalent to a dose of 10 mg Al/day/kg of body weight.

### 2.4. Breast Cancer Induction in Rats

The rats were treated with NMU (N1517-1G Sigma-Aldrich, St. Louis, MO, USA) at doses of 50 mg/kg of body weight administered at 50 and 70 days of age to induce breast adenocarcinoma [30,31].

### 2.5. Biotery Handling Conditions

Biotery handling conditions were as follows: 12 h light/dark cycles, humidity ranging from 40% to 70%, temperature between 18 °C and 22 °C, and ad libitum access to water and food [32].

### 2.6. Diet

Baseline pellet diet containing 23% protein, 1% vitamins, 4% minerals, 4% fiber, 6.5% fat, 0.2% choline bitartrate, 0.2% methionine, other minor dietary components, and starch to make up to a total of 100%, was used. Maximum humidity was 12%. This rat-feed was manufactured by LabDiet, Fort Worth, TX, USA, and marketed by PetFood of México [33].

### 2.7. Sampling

#### 2.7.1. Breast Tissue Samples

Samples were obtained via surgical cuts of the mammary gland of rats anesthetized in a halothane chamber and then euthanized by cervical dislocation to avoid animal suffering, as per the NOM-033-ZOO-1995 and NOM-062-ZOO-1999 Official Mexican Standards, European Medicines Agency (EMEA, Amsterdam, The Netherlands, 2009), and Food and Drug Administration (FDA, Silver Spring, MD, USA, 2014) [34,35,36,37]. After quantifying the aluminum concentration, the samples were used for histopathological evaluation and total RNA extraction to evaluate the genetic expression of *BRCA1* and *SCL11a2*.

#### 2.7.2. Blood Samples

For genotoxicity evaluation, blood was obtained from a tail cut for micronucleus (MN) analysis and individual cells (comet assay) via alkaline electrophoresis.

### 2.8. Determination of Aluminum Concentration in Breast Tissues

Samples were previously digested in a TITAN MPS microwave oven [38]. Briefly, 0.4 ± 0.02 g of breast tissue was weighed and placed in 15 × 2.5 cm Teflon digestion tubes containing 7 mL of concentrated nitric acid (HNO_3_). The digestion conditions were 200 °C, 35 × 10^5^ Pa, and 1600 W for 47 min [39]. The resultant acidic residue was made to a total of 100 mL using deionized water for the subsequent quantification of aluminum concentration.

The determination of aluminum concentration in the standard, certified reference material, and rat breast tissue was performed using atomic absorption spectroscopy with an AAnalyst 400 automated equipment in a graphite furnace absorption atomic spectroscopy (GFAAS) or in the electrothermal absorption atomic spectroscopy (ETAAS) mode, under the operating conditions recommended by the manufacturer [40,41]. The analytical method was previously optimized and its expanded uncertainty for quantifying aluminum by FAAS (flame absorption atomic spectroscopy) and ETAAS was subsequently validated and estimated uncertainty [42,43,44].

### 2.9. Evaluation of Genomic Instability

#### 2.9.1. Histopathological Evaluation

Morphological instability was assessed by preparing histological cuts from rat mammary glands and mounting them on fixed slides using a microtome. The preparations were stained with hematoxylin and eosin and observed under a LEICA DME optical microscope under 40× and 100× magnification [45].

#### 2.9.2. Micronucleus Analysis

To examine MN, three female Sprague Dawley rats weighing 200 ± 20 g were used per treatment group. Peripheral blood sampling was performed according to the present regulations [34,37] at 5, 10, and 15 days of experimental treatment. Briefly, 0.5 mL of peripheral blood was collected in 1.5-mL Eppendorf tubes and three blood smears (extensions) were prepared on clean slides with blood from each rat. The smears were stained with Wright’s stain [46] and MN were subsequently counted in 2000 erythrocytes (1 MN, 2 MN, or >2 MN) [28] under a LEICA DME compound microscope (Buffalo, NY, 14240, USA).

#### 2.9.3. Alkaline Electrophoresis Test in Individual Cells (Comet Assay)

Cell viability was assessed using trypan blue dye, and cells were counted in a Neubauer chamber/hematocytometer [47,48]. The comet test was performed according to Singh’s method [49] at an exploratory level. Briefly, we used three female Sprague Dawley rats from treatments A (−Al/−NMU) and C (+2000Al/−NMU), which were anesthetized in a halothane chamber, to extract 3–4 mL of blood by intracardiac puncture, and these rats were subsequently euthanized by cervical dislocation. A total of 30 µL of whole blood was added to 300 µL of 1% low melting point agarose at 37 °C. A total of 75 µL of this mixture was extracted and placed on a slide pre-coated with a 150-µL layer of 1% regular agarose, which was immediately covered with a coverslip and maintained at 4 °C for 10 min. The coverslip was removed and 75 µL of 1% low melting point agarose was added at 37 °C to create another layer and form a sandwich, which was protected with a coverslip and maintained at 4 °C for 10 min. The cells were subsequently lysed in a lysis solution (2.5 mM NaCl, 1% KOH, 100 mM EDTA, 10 mM Trizma base, 1% Triton X-100, and 10% DMSO) for 1 h at 4 °C. The samples were then placed in a dark electrophoresis chamber containing cold, alkaline running buffer at pH > 13 (300 mM NaOH, 1 nM EDTA, pH adjusted to >13). Samples were stored in the refrigerator for 20 min. The conditions of the electrophoretic run were 25 mV and 300 mA for 20 min.

Following electrophoresis, the slides were removed and washed three times with a neutralization buffer (0.4 mM Tris buffer adjusted to pH 7.5) for 5 min/wash. Finally, the slides were washed twice with anhydrous absolute ethanol for 5 min/wash. Excess alcohol was removed, and the slides were allowed to dry, following which they were then stained with 25 µL ethidium bromide (20 µg/mL in deionized water) and covered with coverslips. All stages of the comet assay were performed under indirect yellow light or in the dark. Comet observations were made under a LEICA DM2500 fluorescence microscope equipped with an excitation filter (515–560 nm) and barrier filter (590 nm). Photographs were captured with a 5-megapixel LEICA model DFC450C digital camera cooled with monochromatic light, with a C-mount adapter. The comet evaluations were performed using the TriTek CometScore software. DNA damage was reported as % Olive Tail Moment (% OTM) [50,51].

#### 2.9.4. Genetic Expression Assay Using RT-qPCR

Chomczynski and Sacchi’s method (1987) [52] was used for total RNA extraction. The mammary gland tissues (0.050 ± 0.008 g) collected from the rats was weighed and used for total RNA extraction using TRIzol. In addition, PolyTron equipment was used to homogenize the samples. Purity and concentration of the total RNA obtained was estimated by absorbance at 260/280 nm using Nanodrop equipment [53], and the integrity was determined by electrophoresis in 1.5% agarose gel under denaturing conditions and then stained with 1.5% ethidium bromide according to Jacobs Protocol (2017) [54]. A reverse transcription reaction was performed with the RNA obtained from each sample using a QuantiTect Reverse Transcription kit to obtain the cDNA, under the operating conditions recommended by the provider (Qiagen, 2016; cat. no. 205311). PCR reactions were conducted in a T100 Thermocycler (BioRad, 2015, Hercules, CA, USA).

On obtaining the cDNA, genetic expressions of *BRCA1* and *SCL11a2* (FAM fluorophore) were evaluated using 30 ng of cDNA and TaqMan genetic expression assay (Applied Biosystems). RT-qPCR reactions were conducted in the StepOneTM v2.3 device using *GAPDH* (VIC fluorophore) as the reference gene (housekeeping gene). Duplex reactions (*BRCA1*/*GAPDH* and *SCL11a2*/*GAPDH*) were performed under the conditions recommended by the provider (Applied Biosystems 2016).

### 2.10. Statistical Analysis

The Kolmogorov–Smirnov test was applied to verify normality of aluminum concentrations in the mammary gland tissues; when these assumptions were not met, the data were analyzed using the Kruskal–Wallis test. Data for the variables that fulfilled the normality assumptions were assessed using one-way analysis of variance using the General Linear Model procedure (GLM ANOVA), where the experimental treatments was the main effect. Regarding the number of MNs, only counts obtained for cells with a single MN were used because no records were available for cells with ≥2 MNs. MN and genotoxicity data were analyzed using GLM ANOVA for a complete block random design. The model included the fixed effects of treatments and sampling times (blocks). Any differences observed between the means were analyzed using the Tukey–Kramer multiple comparison test. Statistical significance was considered at a 0.05 probability for type I error (*p* < 0.05). All data were processed using the NCSS statistical package, version 2007 (Kaysville, UT, USA) The research project was approved by the Commission of Bioethics in Research of the University of Sonora, ex officio CBI-UNISON 1/2015 (Approval date: 9 February 2015).

## 3. Results

### 3.1. Determining Aluminum Concentration in Breast Tissue

Table 1 denotes a significant difference (α = 0.05) between the +2000Al/−NMU treatment and the other experimental treatments, with a value of 38.17 ± 2.49 µg of aluminum/g of mammary gland tissue.

### 3.2. Evaluation of Genomic Instability

#### 3.2.1. Histopathological Evaluation

**Figure 1:**
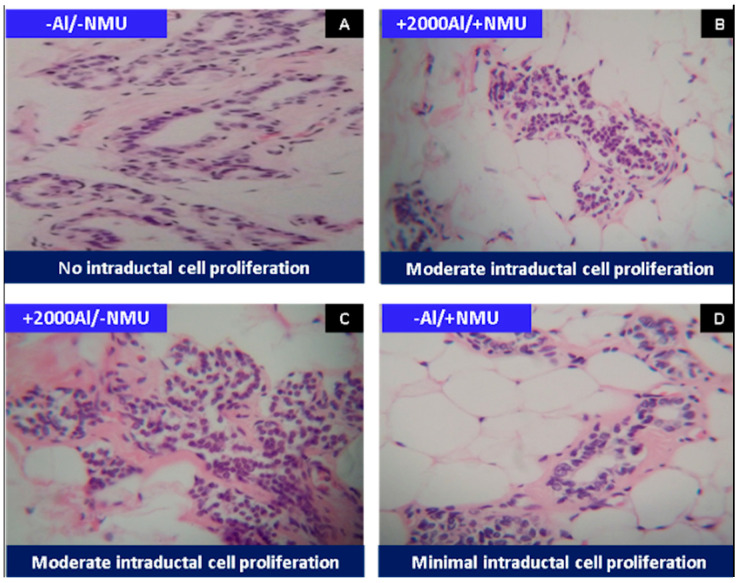
The histological change found in the breast tissue of Sprague Dawley rats is shown with the different treatments used. Hyperplasia of the breast ducts was minimal (**D**) to moderate (**B**,**C**). Normal/negative control (**A**). HEx400.

Histopathological results (Figure 1) showed that the effect of the treatments corresponded to hyperplasia and that there was no evidence of cancer development, similar to the results obtained by some researchers [45,55,56].

#### 3.2.2. Micronucleus Analysis

**Figure 2:**
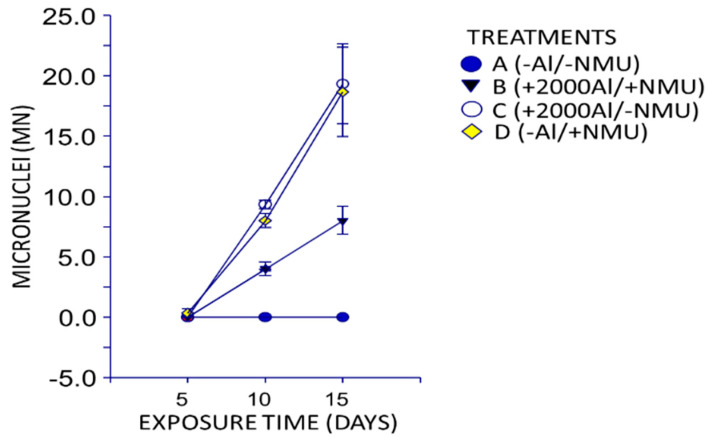
Genotoxicity (1 MN) caused by experimental treatments in the peripheral blood of Sprague Dawley rats at 5, 10, and 15 days of exposure.

MN analysis is a valuable indicator for the partial assessment of genotoxicity via ruptures in the chromosomes [57,58]. The complete MN count is shown in Table 2, which indicates that the presence of MNs was not detected in the negative control (−Al/−MNU) at 5, 10, or 15 days. This is normal to some extent because these rats were not administered aluminum solutions or NMU, a cancer inducing agent. As denoted in Figure 2, an effect from the treatment and exposure time (*p* < 0.05) was observed for counts with 1 MN. Our result suggests that there was no synergistic effect between aluminum (AlCl_3_) and NMU, as previously noted in the results obtained for aluminum concentration in the mammary glands as well as from the histological evaluation of the breast. In the +2000Al/−NMU and −Al/+NMU treatments, a higher number of MN (*p* < 0.05) were observed. Although the average number of MN was higher for the +2000Al/−NMU treatment than for the –Al/+NMU treatment, there was no significant difference (*p* > 0.05) because of the substantial variance among the +2000Al/−NMU treatment replications. Nevertheless, the study proved that the treatment containing only aluminum could independently cause genotoxicity in rats.

In addition, the genotoxic effects started to significantly manifest (*p* < 0.05) on day 10 and showed greater values at day 15 (effect from exposure time), indicating a subacute effect due to aluminum bioconcentration. Additionally, the results revealed that the apparent effect was intermediate in treatments with aluminum and NMU (+2000Al/+NMU).

#### 3.2.3. Alkaline Electrophoresis Test in Individual Cells (Comet Assay)

**Figure 3:**
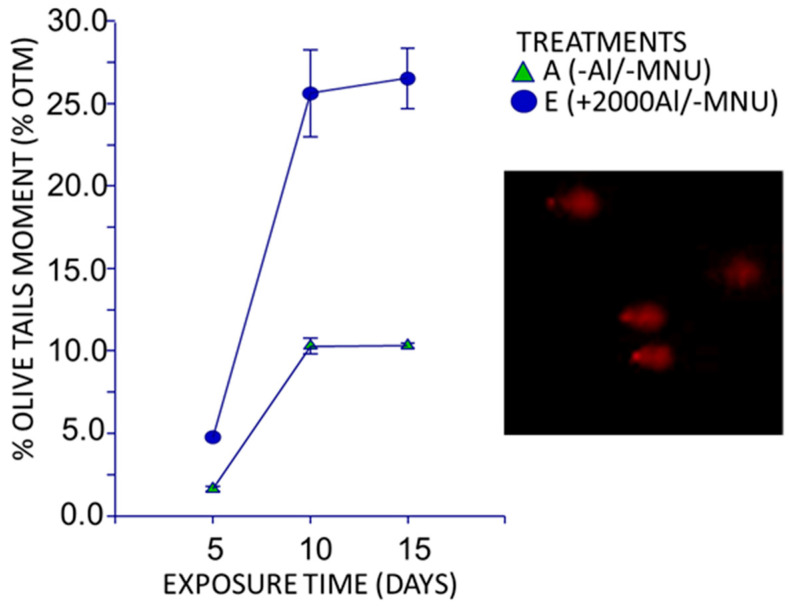
Genotoxic effect caused by the +2000Al/−NMU treatment in the peripheral blood of Sprague Dawley rats at 5, 10, and 15 days of exposure, evaluated by the comet test.

The 96% ± 2% cell viability identified by trypan blue was similar to the results obtained by other researchers [47,48]. This study was conducted at an exploratory level to determine whether the genotoxicity caused by aluminum in the form of AlCl_3_ caused DNA damage or fragmentation in rat leukocytes. Only three rats were used as a negative control (−Al/−NMU) and three rats were subjected to the +2000Al/−NMU experimental treatment, thereby proving that this experimental treatment could independently induce intraductal cell proliferation in mammary glands and lead to a higher number of comet and clouds as exposure time increased. Figure 3 denotes these results, indicating that there was no genotoxic damage at 5, 10, and 15 days of exposure after the −Al/−NMU treatment (negative control). Conversely, no comets were observed in the +2000Al/−NMU experimental treatment, and only nucleoids or unrolled DNA were noted after five days of exposure. The test was consistent with the lack of genotoxicity observed in the formation of MN after five days of exposure. However, genotoxicity was distinctly observed after 10 and 15 days, when comets were observed. Although no significant differences (α = 0.05) were observed in terms of the number of comets and % OTM after 10 and 15 days of aluminum exposure, there were significant differences in terms of cloud formation because there were three and 73 clouds per 100 comets detected at 10 and 15 days of aluminum exposure, respectively.

#### 3.2.4. Genetic Expression Assay Using RT-qPCR

The results of genetic expression obtained for *BRCA1* and *SCL11a2* using RT-qPCR indicate that no experimental evidence demonstrating the expression of both genes was present; therefore, it can be inferred that the product of *BRCA1* does not participate in the DNA damage repair mechanism as observed in the comet test. In addition, it can be determined that the product of *SCL11a2* does not play a role in the aluminum transport process in Sprague Dawley rats under the proposed experimental conditions.

## 4. Discussion

### 4.1. Determining Aluminum Concentration in Breast Tissue

Oogoshi et al. (1994) reported that aluminum concentration was higher in the mammary glands of Sprague Dawley rats compared with other tissues by quantifying the concentration of various metals provided in their diet [59]. Furthermore, these results indicate that there was no synergistic effect of the treatments containing aluminum (AlCl_3_) and NMU (+2000Al/+NMU); therefore, it can be assumed that, among the effects of NMU administration, tumorigenesis does not require aluminum as an essential microelement, or NMU leads to changes in the aluminum transportation mechanism. This effect has been observed when determining that NMU administration in rats modifies the ZnT-1 zinc transporter that causes failure in the zinc transportation mechanism [60,61].

### 4.2. Evaluation of Genomic Instability

#### 4.2.1. Histopathological Evaluation

NMU induces breast cancer in laboratory rats [62,63,64,65,66,67]. However, according to the present study, this compound caused only minimal cell proliferation in the ductal epithelium (−AL/+NMU). Reportedly, adenocarcinomas appeared 140 days after NMU application [68]. Cell proliferation of the ductal epithelium was not observed in treatment A (−Al/−NMU), which was expected because this treatment represents the negative control. The most significant histopathological change caused by treatment C (+2000Al/−NMU) was ductal epithelial cell proliferation compared with the other treatments. A previous histopathological study showed that aluminum in the form of AlCl_3_ can cause liver damage in Sprague Dawley rats when orally administered for 30 days [57].

#### 4.2.2. Micronucleus Analysis

The present study demonstrated that NMU generates MN after 10 days of exposure under the +2000Al/−MNU treatment. A previous study assessed the genotoxicity of aluminum using the MN test and reported that genotoxic agents such as cyclophosphamide generate MN in the peripheral blood of rats within 24 h [69]. On the other hand, Balasubramanyam et al. [28,70] determined that the intraperitoneally administered nanoparticles of Al_2_O_3_ caused an increase in liver MN counts in female Wistar rats. Moreover, they determined that increasing the dosages (500, 1000, or 2000 mg of Al/kg of body weight) increased the number of MN when compared with the control group rats to which cyclophosphamide was administered as a genotoxic agent. This genotoxicity was corroborated by Klien and Godnić-Cvar [71]. Moreover, Türkez et al. [57,72,73] assessed liver genotoxicity caused by AlCl_3_ solutions in Sprague Dawley rats. They observed that a sub-chronic dose of AlCl_3_, intraperitoneally administered for 10 weeks increased the number of MN; however, this effect can be reversed using boric acid or borax. Geyikoglu et al. [58] found that AlCl_3_ at a dose of 3 mg of aluminum/kg of body weight induced genotoxicity by forming MN in the hepatocytes of Sprague Dawley rats when aluminum was intraperitoneally administered for 10 weeks. In addition, Al-Obaidy et al. [74] demonstrated that 10, 15, and 25 mg of AlCl_3_ solutions per kg of body weight intraperitoneally administered to male albino rats (*Rattus norvegicus*) increased the number of MN in the bone marrow, and the number of MN increased with the increase in dose. As the dose increased, the number of cells with MN decreased, and this decrease correlates with an increase in the number of cells that die due to apoptosis [75]. In the present study, we intragastrically (gavage) administered AlCl_3_ solutions and the genotoxic effect observed was similar to that of the intraperitoneally administered aluminum solutions, as previously reported [73,74].

#### 4.2.3. Alkaline Electrophoresis Test in Individual Cells (Comet Assay)

The results obtained in treatment C (+2000Al/−NMU) implies a greater genotoxic effect after 15 days of exposure that is irreversible and considerably greater than the effect of H_2_O_2_ exposure [76,77]. A previous study reported that aluminum in the form of aluminum lactate intragastrically administered for 12 weeks could cause DNA fragmentation in the brain tissue of female Wistar rats when the genotoxic effect was assessed using agarose gel electrophoresis [78]. Using comet and MN tests, correlations were determined between the genotoxic damage caused by aluminum to the DNA of human lymphocytes and an increase in the dosage and exposure time [79]. The advantage of using the alkaline comet assay over the other in vitro methods is that it can detect lesions or damage to single- or double-stranded DNA as well as breaks in labile points in the DNA of single cells [80].

#### 4.2.4. Genetic Expression Assay Using RT-qPCR

*BRCA1* is a genetic marker for breast cancer, and its role in the DNA damage repair mechanism has been demonstrated. Considering that it has undergone mutations, its expression is regulated by epigenetic modification and it is overexpressed in breast cancer [81,82,83]. Furthermore, the product of *SCL11a2* has been identified as a divalent metal transporter gene [84,85,86,87]. The macrophage protein associated with natural resistance, Nramp, which is a product of *SCL11a2*, has recently been linked to aluminum transportation in different organisms [88,89]. Therefore, other molecular biological approaches must be applied in an attempt to establish this genetic relationship. Indeed, other researchers have obtained similar results. For example, cadmium reportedly induces DNA fragmentation and tumorigenesis, but not the expression of genes such as *bcl-x* or *MT-1* in Wistar rat testis and prostate [90]. Rodrigues-Peres et al. [91] found no *ERBB2*, *C-MYC*, and *CCND1* gene instability when assessing the correlation between aluminum and genomic instability in cancerous human breast tissues and healthy tissues. Additionally, as previously reported, the changes were observed at extremely low or nanomolar concentrations of aluminum, with the ability to induce the expression of pro-inflammatory and pro-apoptotic genes. This may be related to some degree of genotoxicity [92,93], as observed by our research group in the present study.

## 5. Conclusions

This study showed that aluminum chloride causes minimum-to-moderate hyperplastic (proliferative) intraductal cell proliferation and genotoxicity. Our results indicate that the BRCA1 product is not involved in the process of DNA damage repair and infer that the SCL11a2 product does not participate in aluminum transport under the proposed experimental conditions.

## Figures and Tables

**Table 1:** Concentration of aluminum (median of µg Al/g of tissue) in the mammary gland of Sprague Dawley rats quantified by graphite furnace absorption atomic spectroscopy (GFAAS).

TREATMENTS *					
Variables	−Al/−NMU	+2000Al/+NMU	+2000Al/−NMU	−Al/+NMU	*p* Value **
n	8	8	8	7	
Mammary gland tissue	11.395 ^a^	12.288 ^a^	38.17 ^b^	17.929 ^a^	0.0001

* = Al/NMU = ± Aluminum/± Nitrosomethylurea; ** = *p* value, Significant *p* ≤ 0.05. (α = 0.05). ^a^ or ^b^ = Different letters means statistical difference.

**Table 2:** Genotoxicity caused by experimental treatments by micronucleus count (MN) in peripheral blood erythrocytes of female Sprague Dawley rats at 5, 10, and 15 days of exposure.

Treatments	5 Days			10 Days			15 Days		
	1 MN	2 MN	>2 MN	1 MN	2 MN	>2 MN	1 MN	2 MN	>2 MN
(A) −Al/−NMU	0	0	0	0	0	0	0	0	0
(B) +2000Al/+NMU	0	0	0	4	0	0	8	0	0
(C) +2000Al/−NMU	0	0	0	9.3	0	0	28.6	2.6	0
(D) −Al/+NMU	1	0	0	8	0	0	18.6	0.6	0
